# First Report on the Frequency and Subtype Distribution of *Blastocystis* sp. in Extensively Reared Holstein-Friesian Cattle from Terceira Island, Azores Archipelago, Portugal

**DOI:** 10.3390/ani15020186

**Published:** 2025-01-11

**Authors:** Sara Gomes-Gonçalves, Alexandra Silva, Guilherme Moreira, Nausicaa Gantois, Rubén Garcia Dominguez, Eric Viscogliosi, Magali Chabé, João R. Mesquita

**Affiliations:** 1 ICBAS—School of Medicine and Biomedical Sciences, Porto University, 4050-313 Porto, Portugalvet.alexandrasilva@gmail.com (A.S.); gmoreiravet@gmail.com (G.M.); 2 Centro Andaluz de Biología del Desarrollo, CSIC, Universidad Pablo de Olavide, 41013 Sevilla, Spain; nausicaa.gantois@pasteur-lille.fr (N.G.); ruben.garcia.d93@gmail.com (R.G.D.); 3 CNRS, Inserm, CHU Lille, Institut Pasteur de Lille, U1019–UMR 9017–CIIL–Centre d’Infection et d’Immunité de Lille, University of Lille, F-59000 Lille, France; eric.viscogliosi@pasteur-lille.fr (E.V.); magali.chabe@univ-lille.fr (M.C.); 4 Centro de Estudos de Ciência Animal (CECA), Instituto de Ciências, Tecnologias e Agroambiente (ICETA), Universidade do Porto (UP), Rua D. Manuel II, Apartado 55142, 4051-401 Porto, Portugal; 5 Associate Laboratory for Animal and Veterinary Science (AL4AnimalS), 1300-477 Lisboa, Portugal

**Keywords:** *Blastocystis* qPCR, zoonotic subtypes, diary cows

## Abstract

This study is the first to report *Blastocystis* sp. in dairy cattle from Terceira Island, part of the Azores Archipelago. *Blastocystis* sp. is an enteric protozoan with high genetic diversity and is associated with both zoonotic and non-zoonotic subtypes (STs). The present survey aimed to fill the gap in knowledge regarding the occurrence and genetic characterization of *Blastocystis* sp. isolates in Holstein-Friesian cattle in this geographical area. A total of 116 stool samples were thus collected from dairy cows, and *Blastocystis* sp. DNA was detected using qPCR. The results revealed a 14.7% occurrence rate (17/116), with seven STs identified including ST3, ST5, ST7, ST10, ST14, ST25, and ST42, with variable frequency. Some of these STs (ST5, ST7, ST10, and ST14) are potentially zoonotic, underscoring their potential public health significance. However, the low frequency of *Blastocystis* sp. in this animal cohort possibly attributed to the extensive grazing system practiced on Terceira Island and the limited presence of zoonotic isolates, suggests a minimal risk of transmission from cattle to humans. This study highlights the importance of further research on the transmission dynamics of *Blastocystis* sp., particularly in regions with varying farming practices, to better understand its epidemiology and zoonotic potential.

## Simple Summary

This study reports the first detection of *Blastocystis* in dairy cattle on Terceira Island, highlighting its genetic diversity and zoonotic potential. The findings suggest a low risk of transmission from cattle to humans due to the region’s grazing practices. Further research is needed to better understand the role of *Blastocystis* in livestock and its implications for public health.

## 1. Introduction

*Blastocystis* sp. is a stramenopile protozoa with significant genetic diversity and an uncertain pathogenicity. It is likely the most common enteric parasite inhabiting the human gut, with over one billion infections worldwide [1]. Its prevalence often exceeds 50% in developing countries, with an average infection rate of 20% in developed nations [2]. The primary transmission route for this protozoan is the fecal-oral route, where *Blastocystis* sp. can be transmitted through the accidental ingestion of environmentally resistant cysts. This can occur via direct contact with infected individuals or through indirect contact with contaminated food or water sources [3]. Although the presence of *Blastocystis* sp. is frequently asymptomatic, its infection has been associated with gastrointestinal illnesses and/or urticaria in numerous clinical cases [4].

In addition to humans, *Blastocystis* sp. has been reported in various animal hosts including non-human primates, birds, rodents, reptiles, amphibians, and other mammals [5]. Phylogenetic analysis based on small-subunit ribosomal RNA (SSU rRNA) gene sequences has evidenced at least 44 legitimate STs, with 17 of these (ST1–ST10, ST12, ST14, ST16, ST23, ST26, ST35, and ST41) capable of infecting humans [6,7,8,9]. ST1–ST4 are the most common in humans worldwide and are considered as anthroponotic, accounting for approximately 90% of infections, despite the presence of other STs [10].

*Blastocystis* sp. is also commonly found in cattle, where at least 16 STs (ST1-ST7, ST10, ST12, ST14, ST17, ST21, ST23-ST26) have been identified, some of which are zoonotic [11]. The latest global systematic review and meta-analysis revealed that the most prevalent *Blastocystis* sp. STs in bovids are ST10 and ST14, with prevalence rates of 32.3% and 22.1%, respectively, which suggests that both STs are well adapted to cattle [11]. The zoonotic importance of these STs are supported as the recent identification of both STs in diverse human cohorts worldwide [12,13,14]. This supports the hypothesis that ruminants, particularly cattle, could serve as significant zoonotic reservoirs for humans, posing a risk to individuals with frequent contact, such as farmers and livestock breeders [15].

Close human–animal interactions increase the risk of zoonotic transmission. In particular, livestock breeders, veterinarians, and individuals in rural agricultural settings are more likely to acquire infections due to their prolonged and frequent exposure to animals, namely ST10 and ST14 associated and prevalent within cattle. [9,16,17,18]. Despite the zoonotic potential of *Blastocystis,* infections in cattle have not been linked to signs of disease, with most cases reported in healthy animals [4,19]. A study by Lee et al. (2018) found a higher prevalence of *Blastocystis* in cattle with normal feces (24.4%) compared to those with diarrhea (5.4%) [20].

Terceira Island, part of the Azores Archipelago in Portugal, covers an area of around 400 km² and operates as an autonomous region with its own political and administrative structures [21]. The Azores Archipelago experiences a stable climate, with temperatures typically ranging from 16 to 25 °C throughout most of the year [22]. Despite its temperate geographical location, this climate is characterized by consistently high relative humidity, resembling conditions found in tropical and subtropical regions [23]. This climate makes this island particularly well-suited for pasture-based dairy production, with milk and dairy products being the primary agricultural outputs. In contrast, the Azores climate differs from the Mediterranean climate that dominates the Portuguese mainland [23].

In 2023, milk production in Portugal totaled 1.996 billion liters, of which 603.43 million liters (30.4%) originated from the Azores Archipelago [24]. Within the archipelago, the islands of São Miguel and Terceira are the leading contributors, producing 405.67 million liters (20.3%) and 149.71 million liters (7.5%) of the total milk production in Portugal, respectively [25].

Despite the importance of the dairy industry in Azores, no studies have been conducted on the presence of *Blastocystis* sp. in cattle from the region, including Terceira Island, the second-largest producer of dairy products in the archipelago. Given the predominance of family-owned farms in the Azores, where close contact between farmers and cattle is common, there is an increasing risk of transmission from cattle harboring potentially zoonotic *Blastocystis* sp. STs. To address this gap, the present study aims to perform the first molecular identification of *Blastocystis* sp. isolates in dairy cattle from Terceira Island.

## 2. Material and Methods

### 2.1. Sampling

**Figure 1:**
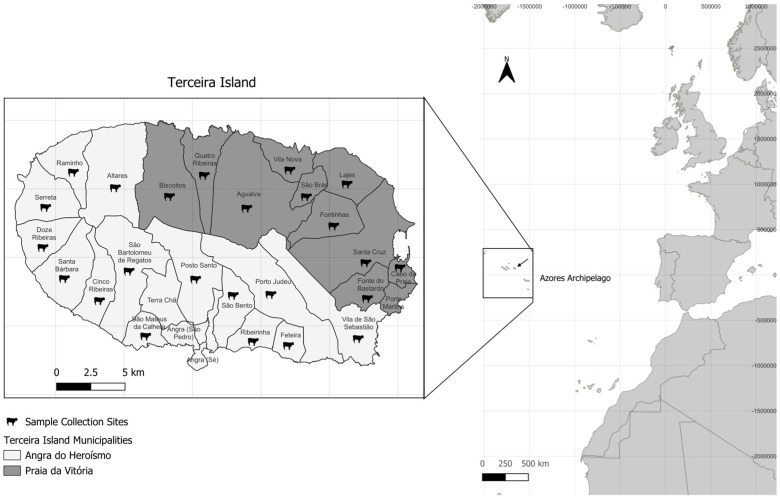
Detailed map of Terceira Island, part of the Azores Archipelago, showing the boundaries of its civil parishes, each labeled with their respective name. The municipalities are shaded in two different tones of grey: The lighter shade represents Angra do Heroísmo, while the darker shade represents Praia da Vitória. Cow icons indicate the locations of the sampling sites for this study. The map scale and coordinates are provided for spatial reference. The construction of the map was performed using QGIS software version 3.36.3.

A total of 116 stool samples were collected from healthy (non-diarreic) adult female Holstein-Friesian dairy cows between October and December 2023. The samples were obtained from 24 cattle farms located in the municipalities of Angra do Heroísmo (70 samples) and Praia da Vitória (46 samples) (Figure 1 and Table 1). The samples were collected either directly from the cows’ recta following transrectal palpation or immediately after defecation. After collection, the samples were refrigerated and transported at 4 °C to the laboratory, where they were stored at −20 °C until DNA extraction.

### 2.2. DNA Extraction and Molecular Detection of Blastocystis sp.

DNA was extracted from approximately 200 mg of stool samples using the NucleoSpin 96 Soil Kit (Macherey-Nagel GmbH & Co. KG, Düren, Germany) following the manufacturer’s instructions. The extracted DNA was eluted in 100 µL of the supplied elution buffer and stored at −20 °C until further analysis. For amplification, 2 µL of purified DNA was subjected to real-time PCR (qPCR) targeting a ~300 bp region of the SSU rRNA gene, employing the primer pair BL18SPPF1 (forward) and BL18SR2PP (reverse), which is specific to the *Blastocystis* genus, as outlined in previous studies [26]. Sequencing was performed on both strands using the same primer pair, by Genoscreen (Lille, France) using the 3730XL DNA Analyzer. The sequences obtained were processed, aligned, and examined using the BioEdit Sequence Alignment Editor software V7.2.5 [27]. The resulting sequences were deposited in the GenBank database with unique accession numbers PQ423101–PQ423109.

### 2.3. Statistical Analysis

The occurrence of *Blastocystis* sp. was assessed by calculating the proportion of positive samples relative to the total number of samples analyzed, along with the corresponding 95% confidence interval (95% CI). Data processing and preliminary analysis were carried out using Microsoft Excel^®^ for Microsoft 365 MSO (Redmond, WA, USA) (version 2312 Build 16.0.17126.20132, 64-bit).

### 2.4. Phylogenetic Analysis and Subtyping of Blastocystis sp. Isolates

Full-length SSU rRNA gene sequences from representatives of the various *Blastocystis* sp. subtypes (STs) and subgroups available at the time of the analysis were retrieved from the GenBank database, serving as the primary reference framework. To improve the phylogenetic resolution for certain isolates, additional sequences were incorporated for ST30, ST21, ST25, and ST14 (5, 6, 8, and 8 additional sequences, respectively). Sequence alignment was performed using MAFFT v7.490, applying the L-INS-i method due to its robustness in aligning diverse sequences. While initial trimming using TrimAl at a threshold of 0.7 was explored, untrimmed alignments were ultimately preferred for maximum-likelihood phylogenetic analysis, as they provided better tree resolution and sample placements. The Maximum Likelihood tree was constructed using IQ-TREE v2.3.6 with 1000 bootstrap replicates, selecting the K2P + I + G4 substitution model to ensure consistency with prior studies [6,7,28]. Moreover, *Proteromonas lacertae*, a commensal flagellate found in reptiles and amphibians, was used as the outgroup due to its close phylogenetic relationship with *Blastocystis* in earlier studies [9,29,30]. The resulting phylogenetic tree was annotated and visualized using the Interactive Tree of Life (iTOL) platform, enabling in-depth representation of phylogenetic relationships [31].

## 3. Results

In the present study, 17 out of 116 samples tested by qPCR were found to be positive for *Blastocystis* sp., of those eight were mixed infection resulting in an overall occurrence of 14.7% (17/116; 95% CI: 8.78–22.42). Except for one sample (PQ423109) from Praia da Vitória (Santa Cruz), all positive samples identified as single infection (*n* = 9) were collected from animals of the Angra do Heroísmo municipality. BLASTn analysis of sequences from these nine samples confirmed the presence of *Blastocystis* sp. and the highest hits are displayed in Table 2.

**Figure 2:**
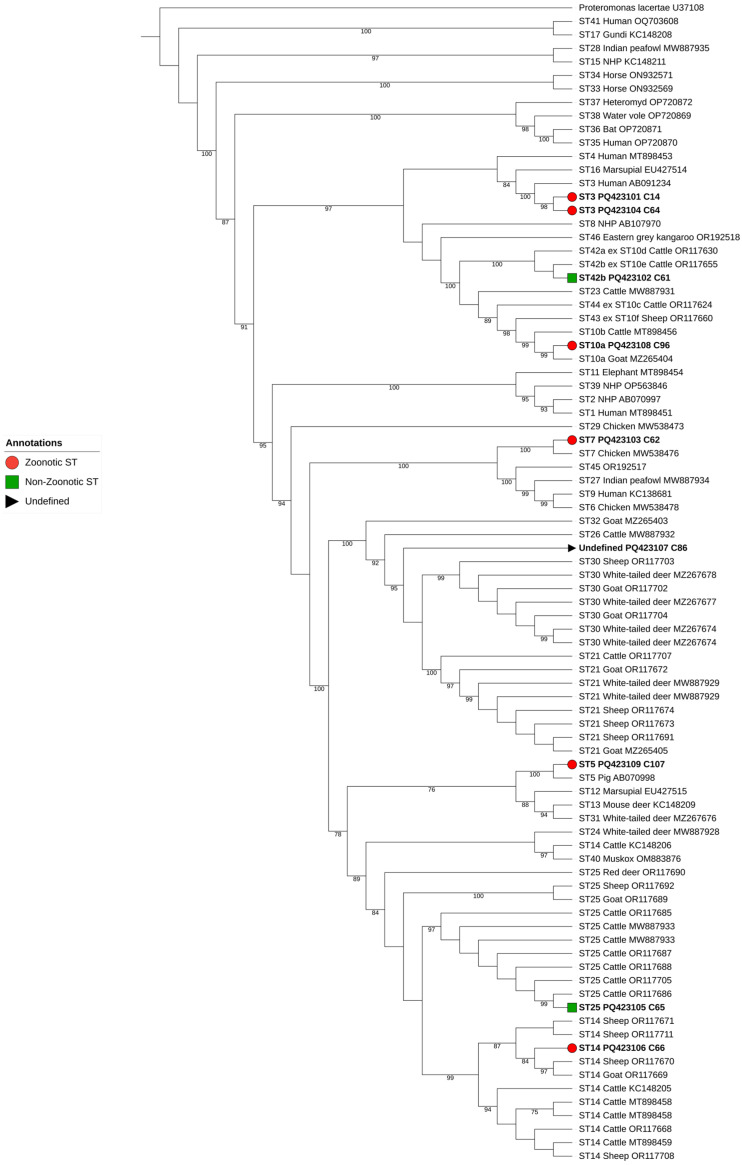
Maximum-likelihood phylogenetic tree of *Blastocystis* sp. STs based on the comparison of SSU rRNA gene sequences. The tree was generated using IQ-TREE with the K2P + I + G4 substitution model and 1000 bootstrap replicates. Zoonotic, non-zoonotic, and undefined isolates are represented by red circles, green squares, and gray triangles, respectively, as indicated in the legend. *Proteromonas lacertae* was used as an outgroup, and bootstraps lower than 75% are not displayed.

The phylogenetic analysis including homologous sequences of all known STs of *Blastocystis* sp., enabled the unambiguous subtyping of 8 out of the 9 sequences obtained in the present study (Figure 2). Seven distinct subtypes were detected: ST3 (two sequences from Altares and Feteira), ST5 (one sequence from Santa Cruz), ST7 (one sequence from Feteira), ST10 (one sequence from Posto Santo), ST14 (one sequence from Feteira), ST25 (one sequence from Feteira), and ST42 (one sequence from São Mateus da Calheta). One sample (sequence PQ423107 from Feteira) could not be assigned to a specific ST but showed some similarity to ST30 and ST21. Most of the identified STs (ST5, ST7, ST10, and ST14) are considered zoonotic and have been reported in humans.

## 4. Discussion

This study represents the first molecular identification and ST distribution of *Blastocystis* sp. isolates colonizing Holstein-Friesian dairy cattle from Terceira Island, part of the Azores Archipelago. It provides new insights into the parasite’s occurrence, genetic diversity, and potential zoonotic risk within this geographically isolated region. Although a diverse range of *Blastocystis* sp. STs was identified, the overall occurrence rate of 14.7% is significantly lower than the global prevalence rate of 24.4% determined worldwide from cattle cohorts [11]. This comparatively low prevalence is also evident when compared to rates reported in other European countries, including Denmark, the UK, Spain, France, and mainland Portugal, as summarized in Table 3.

Notably, with the exception of Italy and Turkey, where lower occurrences were observed, epidemiological surveys conducted in cattle from other European regions reported higher occurrence rates of *Blastocystis* sp. [32,33,34,35,36,37,38]. This lower occurrence on Terceira Island suggests that factors unique to the region, such as environmental conditions and farming practices, may be limiting the transmission of the protozoan, in contrast to the higher occurrence observed on mainland Portugal [38], where environmental conditions and farming practices may be more conducive to its transmission. However, caution is needed when comparing these results with other studies, as differences in sample sizes and methodologies may influence the findings. Regarding the STs identified herein, all sequences corresponding to single infection were successfully classified except for one, which did not cluster with any reference sequence of known ST and was therefore assigned as undefined, as it does not fulfill the criteria for designation as a new ST, as outlined in the proposed guidelines by Maloney, J.G., & Santin, M. (2021) [30]. These criteria include having an almost complete SSU rRNA gene sequence (≥80% of the approximately 1800 bp) and a sequence divergence greater than 4% from any existing ST. In addition to subtype diversity, common mutations, such as single nucleotide polymorphisms (SNPs) and insertions within the SSU rRNA gene, are frequently observed [30]. These variations play a role in defining subtypes and may influence host specificity, zoonotic potential, and virulence, although these relationships are still not fully understood. Some subtypes appear to be more commonly associated with particular hosts, such as ST1–ST4 with humans, ST5 with pigs, ST6 and ST7 with birds, and ST10 and ST14 with cattle [2,4,39]. Subtypes can also modulate the immune response in distinct ways, as exemplified by ST7 and ST1. ST7 has been shown to provoke a pro-inflammatory environment through its interaction with epithelial and dendritic cells, leading to a reduction in beneficial gut bacteria such as *Bifidobacterium longum* and *Lactobacillus brevis* [40,41,42]. Additionally, ST7 increases the production of proteases that can compromise the intestinal barrier, potentially contributing to gut dysfunction [43,44]. In contrast, ST1 is associated with an increased diversity of the gut microbiome and the promotion of an anti-inflammatory state in the intestinal mucosa, highlighting its potential role in maintaining intestinal homeostasis [43,45]. The lower-than-expected occurrence of *Blastocystis* sp. in cattle from Terceira Island contrasts with initial expectations. Indeed, cattle are commonly considered potential reservoirs for zoonotic *Blastocystis* sp. STs, such as ST10 and ST14, which are frequently observed in livestock populations [2]. However, in our study, the predominance of ST3, typically considered an anthroponotic (human-associated) ST, raises interesting possibilities about transmission dynamics. The slightly higher occurrence of ST3 suggests that there may be a greater degree of human-to-animal transmission than initially anticipated, particularly in settings like family-owned farms where closer interaction between humans and their cattle is more common. In such environments, the direct handling of cattle by farmers may contribute to the transmission of anthroponotic STs, such as ST3, from humans to animals. The presence of ST3 in cattle could thus reflect this close, potentially frequent contact, leading to a shift in the assumed source of the infection.

The detection of ST7 in our cohort of bovids further complicates the scenario, as its presence was not found in previous studies in European countries. ST7 is typically associated with birds [2], and its presence in cattle may be associated with possible transmission from ducks, hens, or other avian species. This likely implies a possible cross-species transmission pathway, where birds are contributing to the presence of this avian-adapted ST in the cattle population.

Furthermore, the identification of ST5, a zoonotic subtype commonly found in both humans and animals, adds another layer of complexity to understanding the potential zoonotic risk of *Blastocystis* on Terceira Island. While ST5 was found in this study, its low prevalence suggests a relatively minimal risk of zoonotic transmission from cattle to humans. Nevertheless, it highlights the need for further research into its distribution and zoonotic potential.

However, the overall low occurrence of *Blastocystis* sp., including zoonotic STs, could be attributed to the extensive grazing system practiced on Terceira Island. In this traditional system, cattle are widely dispersed across large pastures, which minimizes close contact between animals and, in turn, lowers the likelihood of parasite transmission within the cattle population. This finding is consistent with a study that reported higher occurrences of *Blastocystis* sp. in backyard cattle compared to those in more extensive farming systems [46], suggesting that farming practices significantly influence transmission rates. The traditional extensive grazing method on Terceira Island likely serves as a natural barrier to the spread of *Blastocystis* sp. whereas more intensive farming systems, in which cattle are confined and kept in closer proximity, tend to facilitate greater transmission of pathogens, including *Blastocystis* sp.

Although the pathogenic role of *Blastocystis* sp. in domestic and wild animals remains unclear, primarily because most studies have focused on non-diarrheic individuals, the primary concern lies in its potential zoonotic risk to humans [38]. In this study, zoonotic ST5, ST7, ST10, and ST14 were detected, but their low occurrence suggests a minimal risk of transmission from cattle to humans. Moreover, the identification of the two non-zoonotic ST25 and ST42b further supports the conclusion that *Blastocystis* sp. poses a limited threat to human health on Terceira Island.

## 5. Conclusions

This study presents the first molecular identification of *Blastocystis* sp. in dairy cattle from Terceira Island. The low prevalence of the parasite, coupled with the limited presence of zoonotic STs, indicates a minimal risk of transmission from cattle to humans in the region. Local farming practices, particularly the extensive grazing systems, are likely to mitigate both the spread of *Blastocystis* among cattle and the potential for human exposure. However, the study is subject to several limitations, including a small sample size, data collection confined to a single season, and the absence of a comparison of different farming practices. These limitations underscore the need for further research on the transmission dynamics of *Blastocystis* sp., to address these gaps and provide a more comprehensive understanding of its zoonotic risks.

## Figures and Tables

**Table 1:** Distribution of the number of samples (*n* = 116) collected from adult female Holstein-Friesian dairy cows across different civil parishes within the municipalities of Angra do Heroísmo and Praia da Vitória. Each entry provides the name of the civil parish and the corresponding number of samples collected from that area.

Municipality	Civil Parish	Number of Samples (*n*)
Angra do Heroísmo	Doze Ribeiras	2
	Cinco Ribeiras	6
	Altares	1
	Feteira	10
	Posto Santo	4
	Porto Judeu	7
	Raminho	5
	Ribeirinha	1
	Santa Bárbara	3
	São Bartolomeu dos Regatos	7
	São Bento	5
	São Mateus da Calheta	5
	São Sebastião	7
	Serreta	7
Praia da Vitória	Agualva	5
	Biscoitos	2
	Cabo da Praia	2
	Fonte do Bastardo	2
	Fontinhas	9
	Lajes	6
	Santa Cruz	7
	São Brás	6
	Vila Nova	5
	Quatro Ribeiras	2
Total	-	116

**Table 2:** Summary of the *Blastocystis* sp. sequences obtained in this study, including the accession numbers from this study, the highest-hit accession numbers from reference BLAST database, the country of origin of the highest hit, percentage identity (%), and the host organism.

Accession Number (This Study)	Accession Number Highest Hit	Country	Perc. Identity (%)	Host
PQ423101	MN326608	China	98.95	Human
PQ423102	OL981854	China	100	Cattle
PQ423103	OP716163	Vietnam	100	Human
PQ423104	MN326608	China	100	Human
PQ423105	OM522176	France	100	Cattle
PQ423106	OM522189	France	99.66	Cattle
PQ423107	PP320637	Egypt	100	Sheep
PQ423108	OL981921	China	100	Cattle
PQ423109	MH883053	Lebanon	100	Cattle

**Table 3:** Prevalence of *Blastocystis* sp. and ST distribution in cattle cohorts from European countries. The same STs as those reported in the present study are highlighted in bold.

Country	% of *Blastocystis* sp. (no. pos/Total no.)	% *Blastocystis* ST (no. pos)	Method	Reference
Italy	7.69% (1/13)	NA (no ST reported)	PCR	[32]
Turkey	11.3% (9/80)	**ST10**: 22.2% (2), **ST14**: 77.8% (7)	PCR	[33]
Denmark	100% (25/25)	**ST5**: 12% (3/25) **ST10**: 48% (12/25)	PCR	[34]
UK	22.6% (7/31),	**ST1**: 3.23% (1/31), **ST5**: 3.23% (1/31) **ST10**: 9.68% (3/31) MI: 6.45% (2/31)	PCR	[35]
Spain	32.14% (108/336)	ST1: 0.30% (1), **ST3**: 0.30% (1), **ST5**: 2.98% (10), **ST10**: 31.55% (106), **ST14**: 19.94% (67), ST21: 20.83% (70), ST23: 11.61% (39), ST24: 8.33% (28), **ST25**: 27.38% (92), ST26: 30.06% (101)	PCR and NGS	[36]
France	54.79% (866/1581)	ST2: 0.13% (2), **ST10**: 2.28% (36), **ST14**: 4.05% (64), Indefinite MI: 3.61% (57)	qPCR	[37]
Portugal	32.18% (28/87)	**ST1**: 1.15% (1), **ST5**: 6.90% (6), **ST10a**: 17.24% (15), ST10b: 2.30% (2), ST13: 1.15% (1), **ST14**: 5.75% (5), ST21: 18.39% (16), ST23: 3.45% (3), ST24a: 2.30% (2), ST24b: 1.15% (1), ST24c: 1.15% (1), **ST25**: 29.89% (26), ST26: 28.74% (25), ST30: 1.15% (1), ST42a: 27.59% (24), **ST42b**: 25.29% (22), ST43: 1.15% (1), ST44: 4.60% (4)	PCR and NGS	[38]

PCR—Conventional polymerase chain reaction; qPCR—Real-time polymerase chain reaction; NGS—Next Generation Sequencing, MI—Mixed Infection.

## Data Availability

The data presented in this study are available on request from the corresponding author.
